# Supplementary material for: Single-Base Methylome Analysis of Sweet Cherry (Prunus avium L.) on Dwarfing Rootstocks Reveals Epigenomic Differences Associated with Scion Dwarfing Conferred by Grafting
Source: Int J Mol Sci. 2024 Oct 16;25(20):11100. doi: 10.3390/ijms252011100 (PMC11508414; doi:10.3390/ijms252011100)
Supplement: Supplementary file 1 [file ijms-25-11100-s001.zip › Table S4. Data description of transcriptome reads for the four group samples with three replicates.pdf]

Table S4. Data description of transcriptome reads for the four group samples with three replicates

Samples	Total clean reads (M)	Q20	Q30	GC context （%）	Tatal mapping (%)	Uniquely mapping (%)
WT_S1	43.44	100%	100%	45.99	85.38	75.66
WT_S2	45.20	100%	100%	45.91	87.46	77.31
WT_S3	43.90	100%	100%	46.08	85.75	75.86
DS_S1	42.15	100%	100%	46.42	89.83	79.61
DS_S2	43.56	100%	100%	45.84	89.38	79.25
DS_S3	43.83	100%	100%	46.04	89.65	79.58
WT_L1	43.59	100%	100%	46.16	85.26	75.07
WT_L2	43.96	100%	100%	45.43	80.05	70.97
WT_L3	43.98	100%	100%	45.68	78.10	69.35
DS_L1	43.94	100%	100%	45.72	86.72	76.80
DS_L2	43.70	100%	100%	46.19	90.69	80.04
DS_L3	43.92	100%	100%	46.34	84.66	74.84
