# Supplementary figures and images for: Single-Base Methylome Analysis of Sweet Cherry (Prunus avium L.) on Dwarfing Rootstocks Reveals Epigenomic Differences Associated with Scion Dwarfing Conferred by Grafting
Source: Int J Mol Sci. 2024 Oct 16;25(20):11100. doi: 10.3390/ijms252011100 (PMC11508414; doi:10.3390/ijms252011100)

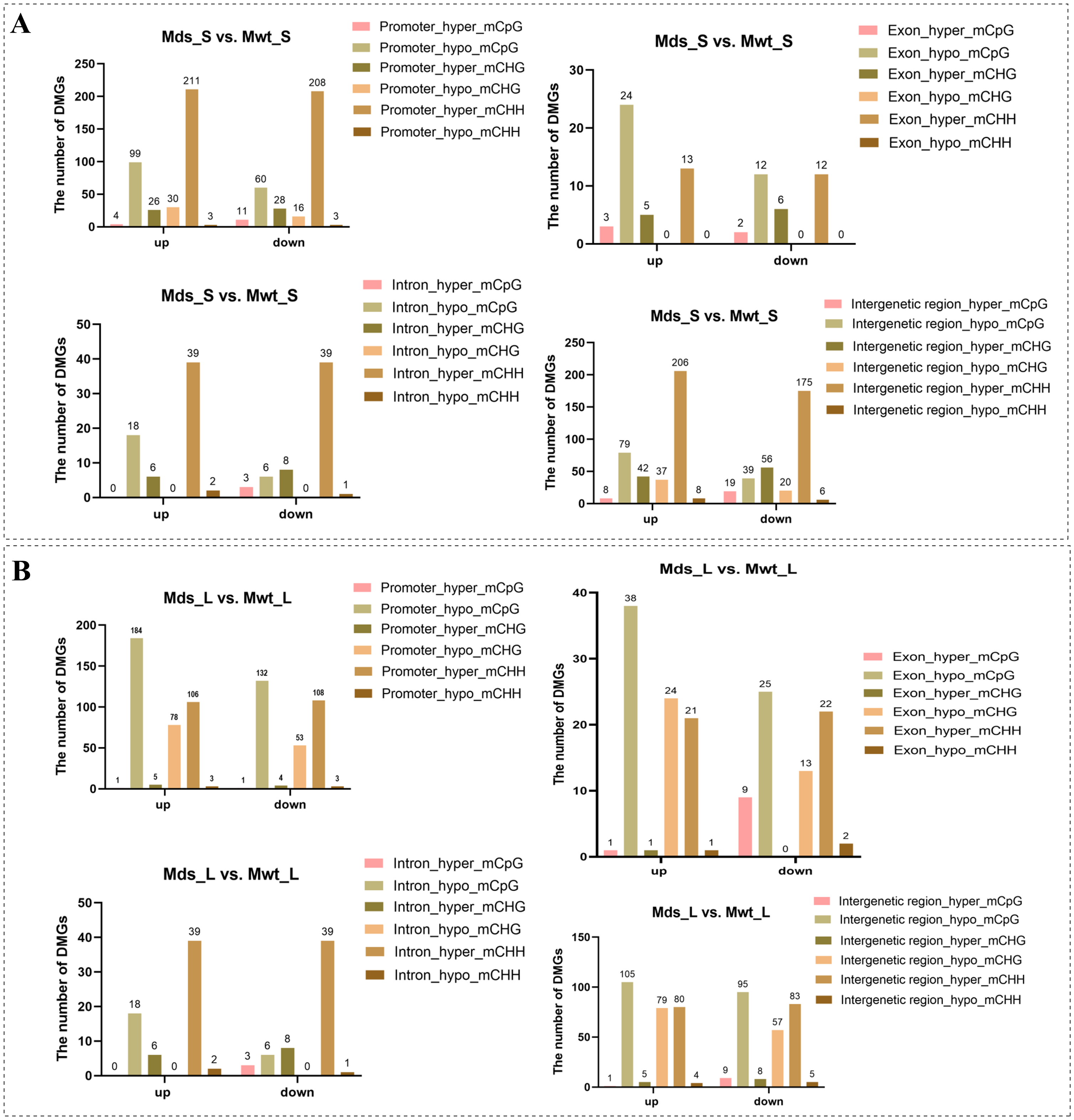

Supplement: Supplementary file 1 [file ijms-25-11100-s001.zip › Figure S1. Statistics of overlapping genes of DMGs and DEGs in shoot tips (A) and leaves (B).tif]
